# Meal-timing patterns and chronic disease prevalence in two representative Austrian studies

**DOI:** 10.1007/s00394-023-03113-z

**Published:** 2023-03-02

**Authors:** Isabel Santonja, Leonie H. Bogl, Jürgen Degenfellner, Gerhard Klösch, Stefan Seidel, Eva Schernhammer, Kyriaki Papantoniou

**Affiliations:** 1 Department of Epidemiology, Center for Public Health, Medical University of Vienna, Vienna, Austria; 2 Department Nutrition and Dietetics, Faculty of Health Professions, Bern University of Applied Sciences, Murtenstrasse 10, 3012 Bern, Switzerland; 3 Department of Neurology, Medical University of Vienna, Vienna, Austria; 4 Channing Division of Network Medicine, Department of Medicine, Brigham and Women’s Hospital and Harvard Medical School, Boston, MA USA; 5 Department of Epidemiology, Harvard T.H. Chan School of Public Health, Boston, MA USA

**Keywords:** Meal-timing, Nighttime fasting, Time-restricted eating, Chrono-nutrition, Cluster analysis

## Abstract

**Purpose:**

This study aimed at describing meal-timing patterns using cluster analysis and explore their association with sleep and chronic diseases, before and during COVID-19 mitigation measures in Austria.

**Methods:**

Information was collected in two surveys in 2017 (*N* = 1004) and 2020 (*N* = 1010) in representative samples of the Austrian population. Timing of main meals, nighttime fasting interval, last-meal-to-bed time, breakfast skipping and eating midpoint were calculated using self-reported information. Cluster analysis was applied to identify meal-timing clusters. Multivariable-adjusted logistic regression models were used to study the association of meal-timing clusters with prevalence of chronic insomnia, depression, diabetes, hypertension, obesity and self-rated bad health status.

**Results:**

In both surveys, median breakfast, lunch and dinner times on weekdays were 7:30, 12:30 and 18:30. One out of four participants skipped breakfast and the median number of eating occasions was 3 in both samples. We observed correlation between the different meal-timing variables. Cluster analysis resulted in the definition of two clusters in each sample (A17 and B17 in 2017, and A20 and B20 in 2020). Clusters A comprised most respondents, with fasting duration of 12–13 h and median eating midpoint between 13:00 and 13:30. Clusters B comprised participants reporting longer fasting intervals and later mealtimes, and a high proportion of breakfast skippers. Chronic insomnia, depression, obesity and self-rated bad health-status were more prevalent in clusters B.

**Conclusions:**

Austrians reported long fasting intervals and low eating frequency. Meal-timing habits were similar before and during the COVID-19-pandemic. Besides individual characteristics of meal-timing, behavioural patterns need to be evaluated in chrono-nutrition epidemiological studies.

**Supplementary Information:**

The online version contains supplementary material available at 10.1007/s00394-023-03113-z.

## Introduction

Circadian misalignment through night shift work has been associated with various chronic disease outcomes [1–6]. Recently, this research has been expanded to identify other sources of circadian disruption in the general population that may result from mistimed exposures, such as sleeping or eating at the “wrong” time [7–9]. More concretely, the adherence to a more diurnal eating pattern has been associated with a lower cancer risk [10–12] and an improvement of metabolic function and weight loss [13]. Moreover, individual aspects associated with a diurnal eating pattern, such as eating breakfast or abstaining from eating at night, have been associated with better sleep quality and longer sleep duration [8]. On the contrary, individuals experience a reduction of sleep duration and an increase of daytime sleepiness during Ramadan, a period of 29–30 days, during which healthy adult Muslims abstain from eating and drinking from dawn to sunset [14]. These observations stress the importance of evaluating timing (in addition to quality, quantity and frequency) of diet in epidemiological studies.

In terms of exposure assessment, most epidemiological studies have characterized meal-timing through questionnaires and have tended to analyse single meal-timing aspects, e.g. dinner time [10] or breakfast skipping [15–20]. However, different meal-timing aspects, such as nighttime fasting interval, interval between the last eating occasion and bed time or breakfast skipping, are interrelated and their effects could overlap. Because of that, Khanna et al. [21] argued that analysing meal-timing behaviours, rather than isolated aspects, using cluster analysis might be a more suitable method of assessing the effects of meal-timing.

Moreover, meal-timing patterns have not been previously described in detail in the Austrian population, and we hypothesized that dietary patterns, especially the eating frequency, could have been modified in Austria as a consequence of the COVID-19 mitigation measures and lockdown, as reported for other populations [22–26]. Furthermore, although evidence starts to emerge that meal-timing might have an influence on health outcomes, little is known in relation to sleep outcomes.

Therefore, the first aim of this study was to describe meal-timing and meal frequency, as well as fasting patterns, and to define predominant meal-timing behaviours in the Austrian population. Secondly, we aimed to compare meal-timing patterns before and during the first COVID-19 mitigation measures and evaluate potential effects of the lockdown on meal-timing habits in Austria. Lastly, we explored the cross-sectional association of meal-timing behaviours and chronic insomnia, chronic disease prevalence and self-rated health-status.

## Methods

### Population and study design

Two online surveys were implemented by Interrogare GmbH − a market research institute based in Germany − , with the aim of eliciting detailed information on sleep habits and its determinants in representative samples of the Austrian general population. We collected information on sleep, daily routines, such as meal-times, and lifestyle characteristics. Participants of both surveys were selected to represent the age (≥ 18 years), sex and county distribution of Austria’s general population. Participation in both surveys was voluntary and anonymous, and informed consent was implied through participation.

The first survey took place in September 2017, included 63 questions and took approximately 30 min to complete. In total, 1004 participants completed the survey. Of those, current night shift workers (*n* = 52) and one participant with missing information in all meal-timing variables were excluded from the present analysis (*n* = 951 participants included in description of meal-timing). Moreover, 27 further participants with missing information on key meal-timing variables were excluded from the cluster and association analysis (*n* = 924 participants included in the cluster analysis).

The second survey was conducted in June 2020, after the first COVID-19 wave and the implementation of mitigation measures in Austria (March 16 to May 1st, 2020). The questionnaire, which included 81 questions and took approximately 20 min to complete, was completed by 1010 participants. Current nightshift workers (*n* = 55) and three additional participants reporting gender other than women or men were excluded for the analysis (*n* = 952 participants included in description of meal timing). Additionally, 82 participants were excluded for the cluster analysis because of insufficient information on meal-timing (*n* = 870 participants included in the cluster analysis).

### Variables

The surveys collected the following meal-timing information during the week and on weekends: timing of breakfast, lunch and dinner (as drop down menu with 1-h intervals, e.g., from 12:00 till 13:00), snacks between meals (“yes”/”no”), snack between breakfast and lunch (“yes”/”no”), snack between lunch and dinner (“yes”/”no”), snack between dinner and breakfast (“yes”/”no”) and timing of last snack of the day (hours, minutes). We used the midpoint of the intervals of the hourly bins (e.g., “12:00–13:00″ was substituted by 12:30) to create pseudocontinuous variables for the time of breakfast, lunch and dinner. The continuous variable *nighttime fasting* was defined as the time elapsed between the last and the first meal of the day. We created two additional continuous variables: *last meal to bed time* (hours) and *eating midpoint* (hours), defined as the midpoint between the first and the last meal of the day. We also generated the discrete variable *number of eating occasions* (ranging from 0 to 6, the maximum number of eating occasions that could be reported in the surveys), which included main meals and snacks, and a dichotomous variable, *breakfast skipping* (“skipping” vs. “eating”). Each variable was calculated independently for weekdays and weekends.

The survey collected detailed information on sleep duration, sleep timing and sleep quality. We used the *3rd edition of the International Classification of Sleep Disorders* (*ICSD-III*) [27] to define *chronic insomnia* (“yes”/”no”), as explained in Weitzer et al. [28]. The surveys also collected information on self-rated health status (“In your opinion: How is your health status in general?”, one answer possible: “very good”/“good”/“moderate”/“bad”/“very bad”) and diagnosed medical conditions (“During the past 12 months, did you have any of the following diseases or conditions?”; multiple answers possible). With this information, we defined the following dichotomous (“yes”/“no”) outcome variables: *depression, diabetes*, *hypertension* and *bad or very vad self-rated health status*. Participants also reported their height and weight, which were used to calculate BMI and define *obesity* [“yes” (BMI ≥ 30 kg/m^2^)/ “no” (BMI < 30 kg/m^2^)].

The survey also collected information on sex and age (“How old are you?”, with respondents asked to fill in their age in the 2017 survey, and to choose one of the following categories in the 2020 survey: “ < 20” / “20–24” / “25–29” / “30–34” / “35–39” / “40–44” / “45–49” / “50–54” / “55–59” / “60–64” / “65–69” / “ ≥ 70”) and other confounders and effect modifiers of interest, i.e. self-rated chronotype (“One hears about “morning” and “evening” types of people. Which ONE of these types do you consider yourself to be?”, one answer possible: “definitely a morning type”/ “rather more a morning than an evening type”/ “rather more an evening than a morning type”/ “definitely an evening type”), marital status (“What is your current marital status?”, one answer possible: “single”/ “married or in a partnership”/ “divorced”/ “widowed”), work status [“What is your current work status?”, multiple answers possible: “(self-) employed full-time” / “(self-) employed part-time” / “retired” / “unemployed” / “student, further training, unpaid work experience” / “disabled” / “in compulsory military or community service” / “household”], alcohol consumption [“How much alcohol do you drink per week? (Please give approximate/average amounts)”, with respondents asked to fill in the number of glasses of beer and wine and shots of liquor/whiskey/gin etc. consumed per week], smoking status (“Do you currently smoke?”, one answer possible: “No, never”/“No, not anymore”/“Yes, I currently smoke”) and history of nightshifts (“Have you ever worked night shifts (schedule including ≥ 3 h of work between 12 pm and 6 am and at least 3 nights/month)?”, one answer possible: “No” / “Yes, in the past” / “Yes, currently”).

### Statistical analysis

Summary statistics [medians and interquartile ranges (IQRs), and frequency (*N* and %)] were used to describe baseline characteristics and meal-timing patterns, independently for each survey. The correlation between numerical meal-timing variables during the week and during the weekend and between different numerical variables in each survey was analysed graphically (with matrix scatter plots) and the significance of correlation was tested using the Pearson correlation coefficient. The association of these variables with *breakfast skipping* was assessed using point biserial correlation. The association between *breakfast skipping* during the week and the weekend was analysed using the χ^2^ test.

Within surveys, cluster analysis was performed to group individuals with similar meal-timing behaviours. *Nighttime fasting*, *last meal to bed time* and *eating midpoint* during the week were standardized and included as indicators. To establish the cluster groups, a combination of hierarchical and non-hierarchical clustering methods was applied. Firstly, we used Ward’s method (hierarchical method) removing univariate outliers (values > 3 SD above or below the mean) and generated the resulting dendrogram, in order to select the optimum number of clusters (two for each survey). Using the initial cluster centres obtained by hierarchical clustering and including also outliers of the variables, an iterative non-hierarchical K-means clustering procedure was applied. The Cohen’s ҡ coefficient for the solutions obtained by hierarchical methods and by non-hierarchical methods (final cluster solution) was 0.96 for the 2017 survey, indicating almost perfect agreement, and 0.77 for the 2020 survey (substantial agreement).

To describe characteristics or predictors of the different cluster groups, as well as participants’ sociodemographic and lifestyle characteristics, summary statistics [medians and interquartile ranges (IQRs), and frequency (*N* and %)] were used. Differences on the indicators between cluster groups were analysed using the Wilcoxon rank-sum test. Within surveys and using the largest cluster as reference category, unconditional logistic regression analysis was performed to study the association of meal-timing behaviours and chronic insomnia, depression, obesity, diabetes, hypertension and self-rated health status. Logistic regression models were used and *odds ratios* (*OR*) with 95% confidence intervals were calculated. Age and sex-adjusted *ORs* (*AORs*) and multivariable-adjusted *ORs* (*MV-ORs*) are presented. In addition to age and sex and based on a directed acyclic graph, we considered the following potential confounders for the multivariable adjusted model: *self-rated chronotype, marital status, work status, alcohol consumption, smoking status* and *history of nightshifts*.

Risk estimates were compared across strata of sex and chronotype profiles (early/late). In sensitivity analyses, only participants without report of heavy alcohol drinking (drinking ≤ 12 standard glasses of alcohol a week) or those who had no history of nightshift were included.

All statistical analyses were performed using *STATA 16*.

## Results

The respondents of both surveys, as well as the sub-samples of participants allocated to cluster groups, had similar baseline characteristics, which are shown in *Suppl. table 1*.

### Meal-timing in the Austrian population in 2017 and 2020

Participants reported similar timing of the main meals during the week (median times of breakfast, lunch and dinner: 7:30, 12:30 and 18:30, respectively) and long nighttime fasting intervals during the week (median: 13 h) in both surveys. Some participants (8.8% in 2017 and 10.1% in 2020) had a snack after dinner around 21:00. The time elapsed between last meal and bedtime during the week was longer in 2020 (6.0 h in 2020 vs. 4.0 h in 2017) and the *number of eating occasions* decreased slightly. During the weekend, participants generally reported later breakfast times and longer fasting intervals compared to weekdays; in 2020, participants also reported later lunch and dinner times during the weekend. Furthermore, eating breakfast was more frequently reported during the weekends than on weekdays (2017: 83.9% vs. 75.4%; 2020: 81.1% vs. 74.9%; Table 1 and Suppl. Figure 1).

**Table 1:** Meal timing, fasting intervals and number of eating occasions in two representative samples of the general Austrian population

	Survey 2017 (*n* = 951)		Survey 2020 (*n* = 952)	
	*N*/*n* (%)	Median (IQR)	N/*n* (%)	Median (IQR)
Weekdays				
Breakfast time	717 (75.4)	7:30 (6:30–8:30)	713 (74.9)	7:30 (6:30–8:30)
Lunch time	814 (85.6)	12:30 (12:30–13:30)	836 (87.8)	12:30 (12:30–13:30)
Dinner time	880 (92.5)	18:30 (18:30–19:30)	864 (90.8)	18:30 (18:30–19:30)
Snack after dinner time	84 (8.8)	21:00 (20:00–21:45)	96 (10.1)	21:01 (20:01–22:01)
Nighttime fasting (*h*)	944 (99.3)	13.0 (12.0–15.0)	887 (93.2)	13.0 (12.0–17.0)
Last meal to bed time (*h*)	924 (97.2)	4.0 (3.0–5.3)	913 (95.9)	6.0 (5.0–7.5)
Number of eating occasions	951 (100.0)	3.0 (3.0–4.0)	952 (100.0)	3.0 (2.0–4.0)
Weekend				
Breakfast time	798 (83.9)	8:30 (7:30–9:30)	772 (81.1)	8:30 (7:30–9:30)
Lunch time	830 (87.3)	12:30 (12:30–13:30)	831 (87.3)	13:30 (12:30–13:30)
Dinner time	872 (91.7)	18:30 (18:30–19:30)	847 (89.0)	19:30 (18:30–19:30)
Snack after dinner time	143 (15.0)	21:00 (20:15–22:00)	135 (14.2)	21:01 (20:01–22:01)
Nighttime fasting (*h*)	938 (98.6)	14.0 (13.0–15.0)	887 (93.2)	14.0 (13.0–16.0)
Last meal to bed time (*h*)	890 (93.6)	4.5 (3.5–5.5)	885 (93.0)	6.5 (5.5–7.5)
Number of eating occasions	951 (100.0)	3.0 (3.0–4.0)	952 (100.0)	3.0 (3.0–4.0)

### Analysis of collinearity and association among meal-timing variables

**Fig. 1:**
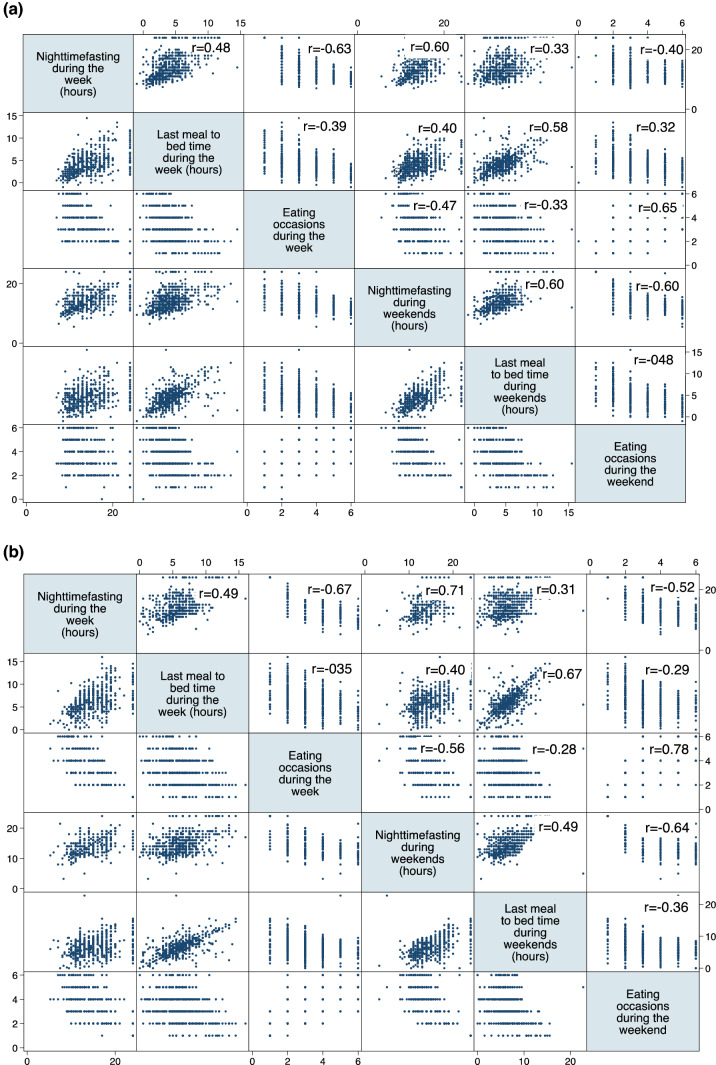
Correlation of meal timing variables in** a** the 2017 survey** b** the 2020 survey. Pearson’s r correlation coefficient is shown in the upper panels of the matrix

We found moderate to strong correlation between individual meal-timing variables (*nighttime fasting*, *last meal to bed time* or *number of eating occasions*) during the week and during the weekend in both surveys (r ≥ 0.58 in 2017 and r ≥ 0.67 in 2020; see *Fig. **1*). *Breakfast skipping* during the week was also correlated with breakfast skipping in the weekend (*p* < 0.001) in both surveys: the majority of those skipping breakfast during the week also reported skipping it during the weekend (2017: *n* = 130/234, 55.6%; 2020: n = 158/239, 66.1%). Because of these associations, we used only meal-timing patterns during the week to generate the cluster solutions and in association analyses.

Furthermore, we found evidence of correlation among the aforementioned meal-timing variables. For example, we found a negative correlation between *nighttime fasting* and the *number of eating occasions*, which was consistent during week and weekend in both surveys, and moderate positive correlation between *nighttime fasting* and *last meal to bed time*, which was particularly strong during the weekends in 2017 (*r* = 0.60). (Fig. 1) In addition, we found a correlation between breakfast patterns and nighttime fasting and number of eating occasions, with participants skipping breakfast reporting longer nighttime fasting periods and eating less frequently (Suppl. Figure 2 and 3 for meal timing patterns during the week; similar results were obtained for the weekend, but are not shown).

### Cluster analysis

In each survey, cluster analysis resulted in the definition of two cluster groups [2017: A17 (*n* = 720; 77.9%) and B17 (*n* = 204; 22.1%); 2020: A20 (*n* = 576; 66.2%) and B20 (*n* = 294; 33.8%)] with different meal-timing patterns (Table 2). Participants in cluster groups B ate later and less frequently, were more likely to skip breakfast and reported longer nighttime fasting intervals and time elapsed from last meal to bed time compared to this in cluster A.

**Table 2:** Distribution of meal timing patterns in cluster groups

	Survey 2017 (n = 924)			Survey 2020 (n = 870)		
	Cluster A17 (*n* = 720) Median (IQR)	Cluster B17 (*n* = 204) Median (IQR)	*p* (cluster differences)^a^	Cluster A20 (*n* = 576) Median (IQR)	Cluster B20 (*n* = 294) Median (IQR)	*p* (cluster differences)^a^
Nighttime fasting* (*h*)	12.0 (11.0–13.3)	18.0 (17.0–20.0)	< 0.001	13.0 (12.0–13.0)	18.0 (16.0–19.0)	< 0.001
Last meal to bed time* (*h*)	4.0 (3.0–5.0)	4.5 (3.5–5.5)	< 0.001	5.5 (4.5–6.5)	7.4 (5.8–9.0)	< 0.001
Breakfast skipping (yes); *N*(%)	46 (6.4)	184 (90.2)	< 0.001	10 (1.74)	175 (59.5)	< 0.001
Eating midpoint* (hh:mm)	13:00 (12:30–14:00)	16:00 (15:00–17:00)	< 0.001	13:30 (12:30–14:00)	15:30 (13:30–16:30)	< 0.001
Number of eating occasions	3.0 (3.0–4.0)	2.0 (2.0–2.5)	< 0.001	3.0 (3.0–4.0)	2.0 (2.0–3.0)	< 0.001

^*^Variables used to generate cluster solution. a. *p*-values calculated using Wilcoxon rank-sum test

The sociodemographic and lifestyle characteristics of the different groups are shown in Table 3. In both surveys, participants in cluster groups B were more likely to be single, divorced or widowed and current smokers, later chronotypes and not to engage in any type of physical activity. Additionally, in 2017, participants in cluster B had a significantly higher BMI. Besides, in 2020, participants in cluster A were more likely to be employed.

**Table 3:** Sociodemographic characteristics across cluster groups in 2017 and 2020

	Survey 2017 (*n* = 924)		Survey 2020 (*n* = 870)	
	Cluster A17 (*n* = 720)	Cluster B17 (*n* = 204)	Cluster A20 (*n* = 576)	Cluster B20 (*n* = 294)
	*N*(%)	*N*(%)	*N*(%)	*N*(%)
Age				
18–24	98 (13.6)	21 (10.3)	71 (12.3)	37 (12.6)
25–34	119 (16.5)	42 (20.6)	117 (20.3)	45 (15.3)
35–44	164 (22.8)	46 (22.5)	131 (22.7)	65 (22.1)
45–54	177 (24.6)	61 (29.9)	138 (24.0)	78 (26.5)
≥ 55	162 (22.5)	34 (16.7)	119 (20.7)	69 (23.5)
Sex (Women)	376 (52.2)	97 (47.5)	298 (51.7)	158 (53.7)
BMI median(IQR)*	24.5 (21.8–27.7)	25.6 (22.2–28.7)	24.2 (21.6–27.7)	24.9 (21.5–29.0)
Education				
High school or less	289 (40.1)	82 (40.2)	197 (34.2)	104 (35.4)
Matura	260 (36.1)	84 (41.2)	206 (35.8)	116 (39.5)
University degree or above	171 (23.8)	38 (18.6)	173 (30.0)	74 (25.2)
Marital status *^,^**				
Single	207 (28.8)	72 (35.3)	175 (30.4)	113 (38.4)
Married/ in a partnership	427 (59.3)	98 (48.0)	352 (61.1)	139 (47.3)
Divorced	75 (10.4)	29 (14.2)	42 (7.3)	35 (11.9)
Widowed	11 (1.5)	5 (2.5)	7 (1.2)	7 (2.4)
Work status **				
Employed full time	363 (50.4)	105 (51.5)	304 (52.8)	145 (49.3)
Employed part time	85 (11.8)	18 (8.8)	79 (13.7)	32 (10.9)
Retired	97 (13.5)	24 (11.8)	61 (10.6)	34 (11.6)
Unemployed and disabled	50 (6.9)	19 (9.3)	38 (6.6)	37 (12.6)
Student, further training…	81 (11.3)	23 (11.3)	70 (12.2)	38 (12.9)
Household	44 (6.1)	15 (7.4)	24 (4.2)	8 (2.7)
Area of residence				
Urban	328 (45.6)	98 (48.0)	286 (49.7)	154 (52.4)
Rural < 50.000 inhabitants	305 (42.4)	84 (41.2)	211 (36.6)	99 (33.7)
Rural > 50.000 *inhabitants*	87 (12.1)	22 (10.8)	79 (13.7)	41 (13.9)
Drinking alcohol				
No standard glasses	285 (39.6)	83 (40.7)	226 (39.2)	113 (38.4)
1–6 standard glasses/week	245 (34.0)	56 (27.5)	259 (45.0)	126 (42.9)
7–12 standard glasses/week	95 (13.2)	36 (17.6)	53 (9.2)	32 (10.9)
> 12 standard glasses/week	95 (13.2)	29 (14.2)	38 (6.6)	23 (7.8)
Smoking status^*, **^				
No, never	333 (46.3)	65 (31.9)	274 (47.6)	110 (37.4)
No, not anymore	204 (28.3)	43 (21.1)	158 (27.4)	74 (25.2)
Yes, I currently smoke	183 (25.4)	96 (47.1)	144 (25.0)	110 (37.4)
Time of physical activity^a,^*^,^**				
No physical activity	247 (34.3)	96 (47.1)	132 (22.9)	90 (30.6)
Before 12 pm	90 (12.5)	14 (6.9)	109 (18.9)	38 (12.9)
12.00–18.00	176 (24.4)	37 (18.1)	144 (25.0)	70 (23.8)
After 18.00	207 (28.8)	57 (27.9)	191 (33.2)	96 (32.7)
Self-rated chronotype*^,^**				
Definitely a morning person	151 (21.0)	33 (16.2)	137 (23.8)	52 (17.7)
Rather a morning person	216 (30.0)	42 (20.6)	160 (27.8)	75 (25.5)
Rather an evening person	238 (33.1)	65 (31.9)	165 (28.7)	85 (28.9)
Definetely an evening person	115 (16.0)	64 (31.4)	114 (19.8)	82 (27.9)
Ever worked on nightshifts	199 (27.6)	51 (25.0)	180 (31.3)	104 (35.4)

Differences in the distribution between cluster groups were evaluated using Pearson’s *χ*^2^

a. In the survey in 2017 information on moderate and vigorous physical activity is available; in 2020 only on vigorous physical activity

^*^p < 0.05 in the survey 2017

^**^p < 0.05 in the survey 2020

### Meal-timing patterns and health status

The self-reported prevalence of common chronic diseases and self-rated health status across cluster groups and survey is shown in Table 4. Subjects in clusters B were more likely to report *chronic insomnia* [AOR (95% CI) = 2.43 (1.46–4.05) in 2017; AOR (95% CI) = 1.70 (1.00–2.90) in 2020], although this effect was reduced after adjusting for lifestyle and sociodemographic confounders [MV-OR (95% CI) = 2.23 (1.29–3.87) in 2017; MV-OR (95% CI) = 1.49 (0.84–2.63) in 2020]. Participants in groups B17 and B20 were also more likely to report having been diagnosed with depression than subjects in A17 and A20, respectively, but risk estimates were only significant in 2020 [MV-OR (95% CI) = 1.55 (1.02–2.36)]. Participants in clusters A were less likely to report obesity, but risk estimates were not significant [MV-OR (95% CI) = 1.18 (0.75–1.85) in 2017 and MV-OR = 1.25 (0.84–1.86) in 2020]. Prevalence of diabetes was similar across surveys and cluster groups, and we did not found any consistent trend for hypertension. Finally, we observed also a higher risk of reporting a bad or very bad health status among subjects in cluster B [AOR (95% CI) = 2.21 (1.27–3.84) in 2017; AOR (95% CI) = 2.86 (1.58–5.20) in 2020], which was reduced after adjusting for confounders [MV-OR (95% CI) = 1.68 (0.92–3.08) in 2017; MVOR (95% CI) = 2.48 (1.29–4.74)].

**Table 4:** Association of meal timing behaviour and self-rated health status and chronic diseases in 2017 and 2020 (*OR: Odds Ratio*, 95%CI: 95% confidence interval)

	Survey 2017 (n = 924)				Survey 2020 (n = 870)			
	Cluster A17 (*n* = 720) *N*(%)	Cluster B17 (*n* = 204) *N*(%)	AOR (95% CI)^a^	MV-OR (95% CI)^b^	Cluster A20 (*n* = 576) *N*(%)	Cluster B20 (*n* = 294) *N*(%)	AOR (95% CI)^a^	MV-OR (95% CI)^b^
Chronic insomnia	43 (6.0)	27 (13.2)	2.43 (1.46–4.05)	2.23 (1.29–3.87)	32 (5.6)	27 (9.2)	1.70 (1.00–2.90)	1.49 (0.84–2.63)
Depression	80 (11.1)	29 (14.2)	1.32 (0.83–2.08)	1.14 (0.70–1.87)	65 (11.3)	56 (19.0)	1.84 (1.25–2.72)	1.55 (1.02–2.36)
Obesity	117 (16.3)	35 (17.2)	1.11 (0.73–1.70)	1.18 (0.75–1.85)	91 (15.8)	60 (20.4)	1.34 (0.92–1.94)	1.25 (0.84–1.86)
Diabetes	33 (4.6)	10 (4.9)	1.20 (0.56–2.54)	1.27 (0.57–2.84)	25 (4.3)	14 (4.8)	1.06 (0.54–2.11)	0.99 (0.47–2.07)
Hypertension	106 (14.7)	25 (12.3)	0.86 (0.53–1.41)	0.81 (0.48–1.36)	77 (13.4)	49 (16.7)	1.26 (0.84–1.89)	1.10 (0.71–1.70)
Bad or very bad self-rated health status	38 (5.3)	22 (10.8)	2.21 (1.27–3.84)	1.68 (0.92–3.08)	20 (3.5)	28 (9.5)	2.86 (1.58–5.20)	2.48 (1.29–4.74)

ORs calculated using unconditional logistic regression

a. AOR = Adjusted OR. Adjusted for age and sex

b.MV-OR = multivariable-adjusted OR. Adjusted for age, sex, self-rated chronotype, marital status, work status, alcohol consumption, smoking status and history of nightshifts

Stratification resulted in low numbers and, thus, only a model adjusted for age and sex was calculated (*Suppl. table 2* and *3*). Some differences by sex categories were observed (*Suppl. Table 2*). The increased risk of chronic insomnia was stronger among men [AOR (95% CI) = 2.63 (1.24–5.62) in 2017 and AOR (95% CI) = 2.78 (1.22–6.38) in 2020] than among women [AOR (95% CI) = 2.41 (1.20–4.86) in 2017 and AOR (95% CI) = 1.17 (0.57–2.41) in 2020]. In turn, the effect of meal-timing on *depression* was only significant among women [AOR (95% CI) = 1.87 (1.01–3.49) in 2017 and AOR (95% CI) = 1.91 (1.15–3.19) in 2020] and the effect on self-rated health status was also stronger among them [AOR (95% CI) = 3.16 (1.44–6.94) in 2017 and AOR (95% CI) = 3.02 (1.27–7.18) in 2020]. In the analysis stratified by chronotypes (*Suppl. Table 3*), the higher risk of depression in cluster groups B was stronger among subjects with early chronotypes [AOR (95% CI) = 2.01 (0.95–4.22) in 2017 and AOR (95% CI) = 1.98 (1.12–3.50) in 2020], while the effect on health-status was stronger among those with late chronotype [AOR (95% CI) = 2.51 (1.22–5.17) in 2017 and AOR (95% CI) = 3.35 (1.51–7.43) in 2020].

Results from analysis restricting to never night shift workers (Suppl. table 4) and to participants without report of heavy alcohol drinking (Suppl. table 5) were similar to the main analysis.

## Discussion

In this study, we provide a description of meal-timing patterns in the Austrian population in 2017 (prepandemic) and in 2020 (during the first COVID-19 mitigation measures), and explored the associations between meal-timing patterns and health outcomes. In both surveys, participants reported eating between 7:30 (median breakfast time) and 18:30 (median dinnertime) or, for those who reported having a snack after dinner, around 21:00 (median snack after dinner time) during the week. Lunch was eaten around 12:30 (median) and breakfast was skipped by about 25% of Austrians on weekdays. In 2020, all main meals were eaten later on weekends than during the week. We observed long nighttime fasting periods and a frequency of 3 eating occasions a day in both surveys. We found correlation between meal-timing variables, and therefore, performed cluster analysis in each survey to group participants according to different meal timing behavioural patterns. The results of the cluster analysis were similar in 2017 and 2020: one group (A17 or A20) was formed by the majority of the participants, who reported long fasting periods and early mealtimes; the remaining participants comprised groups B17 and B20, characterized by even longer fasting intervals, later mealtimes and a high proportion of breakfast skippers.

Huseinovic et al. [29] described a north–south gradient of meal-times in Europe, with Scandinavian countries eating earlier and Mediterranean countries later. According to this, meal-times in the Austrian population are similar to those reported for countries like Germany or Denmark. On the other hand, differences in meal-timing between countries could also be explained by differences in longitude between countries within the same time zone. Austria is located in the extreme east of the Central European time zone, with earlier sunrise and sunset throughout the year. This could explain why Austrian meal-times are earlier compared to countries that are in the same latitude but situated in the western extreme of their time zone and, thus, experience later sun time (e.g. France, Spain). Huseinovic et al. [29] also showed that countries in Central and Northern Europe tend to consume more calories later in the day than earlier on. This is in line with our finding that around one quarter of the Austrians skip breakfast during the week and around one fifth during weekends. However, this proportion is strikingly higher than the one described in a cross-sectional study in Poland during the COVID-19 mitigation measures [22]. In this study, with a younger study population than ours and an under-representation of men, and in which individuals working on a regular basis during the lockdown were excluded, only 1.2% of the participants reported never eating breakfast.

The *number of eating occasions* (median = 3 in both surveys) reported in our samples was rather low, compared to the numbers described in other countries. Based on self-reported data, Americans have about 5.6 meals a day [30] and a study carried out in five European countries reported an eating frequency that ranked between 4.3 (France) and 7.1 (The Netherlands) eating occasions/day [31]. According to Huseinovic et al. [29], people living in Northern and Central Europe eat more frequently than those in southern, Mediterranean countries do. The low eating frequency reported in our study might be partly explained by the way we assessed exposure. In our survey, information on maximal 6 eating episodes was obtained (3 main meals and 3 snacks) and participants could not report having two or more snacks between two main meals or after dinner, which could have resulted in underreporting of eating occasions.

Suprisingly, meal-timing and frequency during the COVID-19 mitigation measures was very similar to in 2017. The results on eating frequency oppose studies from other countries that have described an increase in snacking frequency during lockdowns [23, 25]. In a survey conducted in a representative sample of the population of Jordan [24], participants were more likely to have breakfast, lunch and dinner during the lockdown than before mitigation measures. Furthermore, a study conducted among students in Peru [26] found delays in meal-times and eating-midpoint and a reduction of the nighttime fasting interval after 12 weeks of lockdown measures. Some of these inconsistent findings might be due to differences in the formulation of the meal-timing questions and the fact that we compared the results of the 2020 survey with prepandemic information collected in a different population sample and, thus, intra-individual comparisons could not be performed. Besides, lockdown measures differed among countries and these differences might also explain the discrepancies with our findings.

We also explored the interrelationship between meal-timing and frequency variables and observed moderate correlation between the different variables considered in our study. The association between longer nighttime fasting and skipping breakfast or having an earlier last meal of the day is evident, and this interdependence of meal-timing aspects has also been described previously [32, 33]. Therefore, there has been a claim for analysis focusing on behaviours rather than isolated aspects [21]. This was our motivation for using cluster analysis to group participants according to their combined patterns of meal-timing. Indeed, by doing so, we identified two distinct meal-timing clusters in both surveys that were associated with different insomnia and chronic disease prevalences.

Our exploratory analysis across clusters revealed that insomnia symptoms were more prevalent among participants in groups B17 and B20. Prevalence of depression was also consistently higher in clusters B than A. Besides, in both surveys, participants with later mealtimes and longer fasting periods were slightly more likely to report being obese. There was also an association of meal-timing behaviours with self-rated health status, which was weaker in 2017 than in 2020. These results suggest that longer fasting intervals are not beneficial under all circumstances and that, in everyday life, longer fasting intervals might be the consequence of skipping mealtimes earlier in the day, such as breakfast. Therefore, some of the beneficial health effects usually attributed to fasting in controlled conditions might be reduced or reverted in real-life settings.

Althoug any study had evaluated meal-timing behavioural clusters in relation to chronic diseases before, several studies have analysed the association between individual aspects of meal-timing and chronic disease outcomes. Traditionally, eating breakfast and having several smaller meals in a day have been considered healthy behaviours [34]. Indeed, recent meta-analyses of observational studies concluded that skipping breakfast might be associated with higher risk of diabetes [35], depression [36], overweight and obesity [37]. However, this last assumption has been challenged by a meta-analysis of randomized trials, in which breakfast skipping was associated with modest weight loss [38]. Moreover, Schwingshackl et al. [39] showed that the evidence from randomized control trials did not support the belief that a higher meal frequency contributes to weight control. Concurrently, there has been a shift of paradigm and evidence from experimental studies is emerging, showing prolonged fasting intervals (≥ 12 h), like the ones reported in our samples, might be a successful strategy to reduce body weight [40] or blood pressure [41]. On top of that, not only the duration of the fasting window, but also aligning the eating window with the day and the fasting window with the night might contribute to better health outcomes, through the synchronization of central and peripheral clocks [34]. Eating during the dark phase might also negatively affect sleep, as signalled by a meta-analysis of studies conducted during Ramadan [14]. However, it is unclear how all these interrelated meal-timing aspects interact with each other and affect human health, and studies analysing the combined effects of fasting and meal-frequency and timing are lacking.

To our knowledge, this is the first study to analyse meal-timing as a behaviour, rather than isolated aspects of meal-timing, using cluster analysis to characterize meal-timing behaviours in real-life settings. It is also the first study to describe meal-timing in a representative sample of the Austrian population. Another strength is that our results are consistent in the two samples analysed. The inclusion of two identical surveys administered at two time points in representative samples of Austrians, allowed the comparison of data obtained before and during the pandemic. The analysis was carefully adjusted for a wide range of potential confounders. An additional strength is the definition used for chronic insomnia, which was based on criteria suggested by the *ISCD-III* [27]. Our analysis also has several limitations. First, as this is a cross sectional study, we are unable to comment on the direction of the described associations. Second, we performed several statistical analysis and, therefore, our results on the association between meal-timing and health outcomes are subject to multiple testing errors. This was an exploratory analysis and results should be interpreted with caution. Third, our surveys did not collect information on food content, liquid intake or diet type. Fourth, we might have underestimated the number of eating occasions, as a maximum of one snack could be reported between two main meals in our surveys. Last, information on meal-times was self-reported. Studies using objective meal-timing measures show that human eating patterns are erratic and not well captured through self-reports [42, 43], so under- or over-reporting of eating occasions cannot be discarded in our study and the long fasting intervals observed in our sample might be a consequence of this. Moreover, the questions used for exposure assessment had not been validated against objective measures, as, to our knowledge, at the time the surveys were conducted, no validated meal-timing questionnaire existed. This lack of validated tools also limits the comparability of our results with other studies. Recently, some studies have been published showing moderate agreement between short meal-timing questionnaires and the Automated Self-Administered 24-h recall (ASA24®) Dietary Assessment tool [44] and prospective food records [45]. Future studies on meal-timing with improved and validated reporting methods are, therefore, necessary to confirm our results.

In conclusion, our results suggest that Austrians have earlier meal-times, longer fasting intervals and lower eating frequency than most European countries, although these results might be partly due to an under-reporting of eating occasions. In Austria, meal-timing habits barely changed during the first COVID-19 mitigation measures. Individual meal-timing aspects were highly correlated between them and cluster analysis revealed two well-differentiated groups with different meal-timing behaviours in both surveys. Future epidemiologic studies with improved reporting (e.g. validated questionnaires and objective measures of meal-timing) and analytical methods (e.g. cluster analysis of behavioural patterns) are warranted to evaluate the impact of meal-timing on chronic disease risk.

## Supplementary Information

Below is the link to the electronic supplementary material.

Supplementary file1 (DOCX 234 KB)

## Data Availability

The data that support the findings of this study are available from the corresponding author [IS] and principal investigators of the study (KP, ES) upon reasonable request.

## References

[1] Ward EM, Germolec D, Kogevinas M, McCormick D, Vermeulen R, Anisimov VN, Aronson KJ, Bhatti P, Cocco P, Costa G, Dorman DC, Fu L, Garde AH, Guénel P, Hansen J, Härmä MI, Kawai K, Khizkhin EA, Knutsson A, Lévi F, Moreno CRC, Pukkala E, Schernhammer E, Travis R, Waters M, Yakubovskaya M, Zeeb H, Zhu Y, Zienolddiny S, Grosse Y, Hall AL, Benbrahim-Tallaa L, Girschik J, Bouvard V, El Ghissassi F, Turner MC, Diver WR, Herceg Z, Olson N, Rowan EG, Rumgay H, Guyton KZ, Schubauer-Berigan MK (2019). Carcinogenicity of night shift work. Lancet Oncol.

[2] Vetter C, Devore EE, Wegrzyn LR, Massa J, Speizer FE, Kawachi I, Rosner B, Stampfer MJ, Schernhammer ES (2016). Association between rotating night shift work and risk of coronary heart disease among women. JAMA.

[3] Wang D, Ruan W, Chen Z, Peng Y, Li W (2018). Shift work and risk of cardiovascular disease morbidity and mortality: A dose-response meta-analysis of cohort studies. Eur J Prev Cardiol.

[4] Liu Q, Shi J, Duan P, Liu B, Li T, Wang C, Li H, Yang T, Gan Y, Wang X, Cao S, Lu Z (2018). Is shift work associated with a higher risk of overweight or obesity? A systematic review of observational studies with meta-analysis. Int J Epidemiol.

[5] Gan Y, Yang C, Tong X, Sun H, Cong Y, Yin X, Li L, Cao S, Dong X, Gong Y, Shi O, Deng J, Bi H, Lu Z (2015). Shift work and diabetes mellitus: a meta-analysis of observational studies. Occup Environ Med.

[6] Strohmaier S, Devore EE, Zhang Y, Schernhammer ES (2018). A review of data of findings on night shift work and the development of DM and CVD Events: a synthesis of the proposed molecular mechanisms. Curr Diabetes Rep.

[7] Bonmati-Carrion M, Arguelles-Prieto R, Martinez-Madrid M, Reiter R, Hardeland R, Rol M, Madrid J (2014). Protecting the melatonin rhythm through circadian healthy light exposure. Int J Mol Sci.

[8] Pot GK (2018). Sleep and dietary habits in the urban environment: the role of chrono-nutrition. Proc Nutr Soc.

[9] Castro MA, Garcez MR, Pereira JL, Fisberg RM (2019). Eating behaviours and dietary intake associations with self-reported sleep duration of free-living Brazilian adults. Appetite.

[10] Kogevinas M, Espinosa A, Castello A, Gomez-Acebo I, Guevara M, Martin V, Amiano P, Alguacil J, Peiro R, Moreno V, Costas L, Fernandez-Tardon G, Jimenez JJ, Marcos-Gragera R, Perez-Gomez B, Llorca J, Moreno-Iribas C, Fernandez-Villa T, Oribe M, Aragones N, Papantoniou K, Pollan M, Castano-Vinyals G, Romaguera D (2018). Effect of mistimed eating patterns on breast and prostate cancer risk (MCC-Spain Study). Int J Cancer.

[11] Srour B, Plancoulaine S, Andreeva VA, Fassier P, Julia C, Galan P, Hercberg S, Deschasaux M, Latino-Martel P, Touvier M (2018). Circadian nutritional behaviours and cancer risk: New insights from the NutriNet-santé prospective cohort study: Disclaimers. Int J Cancer.

[12] Palomar-Cros A, Espinosa A, Straif K, Pérez-Gómez B, Papantoniou K, Gómez-Acebo I, Molina-Barceló A, Olmedo-Requena R, Alguacil J, Fernández-Tardón G, Casabonne D, Aragonés N, Castaño-Vinyals G, Pollán M, Romaguera D, Kogevinas M (2021). The Association of nighttime fasting duration and prostate cancer risk: results from the multicase-control (MCC) Study in Spain. Nutrients.

[13] Allison KC, Goel N (2018). Timing of eating in adults across the weight spectrum: Metabolic factors and potential circadian mechanisms. Physiol Behav.

[14] Faris MAE, Jahrami HA, Alhayki FA, Alkhawaja NA, Ali AM, Aljeeb SH, Abdulghani IH, BaHammam AS (2020). Effect of diurnal fasting on sleep during Ramadan: a systematic review and meta-analysis. Sleep Breath.

[15] Lee SA, Park EC, Ju YJ, Lee TH, Han E, Kim TH (2017). Breakfast consumption and depressive mood: A focus on socioeconomic status. Appetite.

[16] Lee YS, Kim TH (2019). Household food insecurity and breakfast skipping: Their association with depressive symptoms. Psychiatry Res.

[17] Miki T, Eguchi M, Kuwahara K, Kochi T, Akter S, Kashino I, Hu H, Kurotani K, Kabe I, Kawakami N, Nanri A, Mizoue T (2019). Breakfast consumption and the risk of depressive symptoms: The Furukawa Nutrition and Health Study. Psychiatry Res.

[18] Ren Z, Cao J, Cheng P, Shi D, Cao B, Yang G, Liang S, Du F, Su N, Yu M, Zhang C, Wang Y, Liang R, Guo L, Peng L (2020). Association between breakfast consumption and depressive symptoms among chinese college students: a cross-sectional and Prospective Cohort Study. Int J Environ Res Pub Health.

[19] Zhu Z, Cui Y, Gong Q, Huang C, Guo F, Li W, Zhang W, Chen Y, Cheng X, Wang Y (2019). Frequency of breakfast consumption is inversely associated with the risk of depressive symptoms among Chinese university students: A cross-sectional study. PLoS One.

[20] Joo HJ, Kim GR, Park EC, Jang SI (2020). Association between Frequency of breakfast consumption and insulin resistance using triglyceride-glucose index: A Cross-Sectional Study of the Korea National Health and nutrition examination survey (2016–2018). Int J Environ Res Pub Health.

[21] Khanna N, Eicher-Miller HA, Boushey CJ, Gelfand SB, Delp EJ (2011). Temporal Dietary patterns using Kernel k-means clustering. ISM.

[22] Sidor A, Rzymski P (2020). Dietary choices and habits during COVID-19 Lockdown: experience from Poland. Nutrients.

[23] Zeigler Z (2021). COVID-19 Self-quarantine and weight gain risk factors in adults. Curr Obes Rep.

[24] Al-Domi H, Al-Dalaeen A, Al-Rosan S, Batarseh N, Nawaiseh H (2021). Healthy nutritional behavior during COVID-19 lockdown: A cross-sectional study. Clin Nutr ESPEN.

[25] Kriaucioniene V, Bagdonaviciene L, Rodríguez-Pérez C, Petkeviciene J (2020). Associations between changes in health behaviours and body weight during the COVID-19 Quarantine in Lithuania: The Lithuanian COVIDiet Study. Nutrients.

[26] Baquerizo-Sedano L, Chaquila JA, Aguilar L, Ordovás JM, González-Muniesa P, Garaulet M (2022). Anti-COVID-19 measures threaten our healthy body weight: Changes in sleep and external synchronizers of circadian clocks during confinement. Clin Nutr.

[27] American Academy of Sleep M (2014) International classification of sleep disorders.

[28] Weitzer J, Santonja I, Degenfellner J, Yang L, Jordakieva G, Crevenna R, Seidel S, Klösch G, Schernhammer E, Papantoniou K (2021). Sleep complaints in former and current night shift workers: findings from two cross-sectional studies in Austria. Chronobiol Int.

[29] Huseinovic E, Winkvist A, Freisling H, Slimani N, Boeing H, Buckland G, Schwingshackl L, Olsen A, Tjonneland A, Stepien M, Boutron-Ruault MC, Mancini F, Artaud F, Kuhn T, Katzke V, Trichopoulou A, Naska A, Orfanos P, Tumino R, Masala G, Krogh V, Santucci de Magistris M, Ocke MC, Brustad M, Jensen TE, Skeie G, Rodriguez-Barranco M, Huerta JM, Ardanaz E, Quiros JR, Jakszyn P, Sonestedt E, Ericson U, Wennberg M, Key TJ, Aune D, Riboli E, Weiderpass E, Berteus Forslund H (2019). Timing of eating across ten European countries - results from the European Prospective Investigation into Cancer and Nutrition (EPIC) calibration study. Public Health Nutr.

[30] Hunt KJ, St Peter JV, Malek AM, Vrana-Diaz C, Marriott BP, Greenberg D (2020). Daily eating frequency in US Adults: associations with low-calorie sweeteners, body mass index, and nutrient intake (NHANES 2007–2016). Nutrients.

[31] Park MK, Freisling H, Huseinovic E, Winkvist A, Huybrechts I, Crispim SP, de Vries JHM, Geelen A, Niekerk M, van Rossum C, Slimani N (2018). Comparison of meal patterns across five European countries using standardized 24-h recall (GloboDiet) data from the EFCOVAL project. Eur J Nutr.

[32] Ruddick-Collins LC, Morgan PJ, Johnstone AM (2020). Mealtime: A circadian disruptor and determinant of energy balance?. J Neuroendocrinol.

[33] Kant AK, Graubard BI (2015). Within-person comparison of eating behaviors, time of eating, and dietary intake on days with and without breakfast: NHANES 2005–2010. Am J Clin Nutr.

[34] Paoli A, Tinsley G, Bianco A, Moro T (2019). The Influence of Meal Frequency and Timing on Health in Humans: The Role of Fasting. Nutrients.

[35] Ballon A, Neuenschwander M, Schlesinger S (2018). Breakfast skipping is associated with increased risk of type 2 Diabetes among Adults: a systematic review and meta-analysis of Prospective Cohort Studies. J Nutr.

[36] Zahedi H, Djalalinia S, Sadeghi O, Zare Garizi F, Asayesh H, Payab M, Zarei M, Qorbani M (2022). Breakfast consumption and mental health: a systematic review and meta-analysis of observational studies. Nutr Neurosci.

[37] Ma X, Chen Q, Pu Y, Guo M, Jiang Z, Huang W, Long Y, Xu Y (2020). Skipping breakfast is associated with overweight and obesity: A systematic review and meta-analysis. Obes Res Clin Pract.

[38] Bonnet JP, Cardel MI, Cellini J, Hu FB, Guasch-Ferre M (2020). Breakfast Skipping, body composition, and cardiometabolic risk: a systematic review and meta-analysis of randomized trials. Obesity (Silver Spring).

[39] Schwingshackl L, Nitschke K, Zähringer J, Bischoff K, Lohner S, Torbahn G, Schlesinger S, Schmucker C, Meerpohl JJ (2020). Impact of meal frequency on anthropometric outcomes: a systematic review and network meta-analysis of randomized controlled trials. Adv Nutr.

[40] Liu L, Chen W, Wu D, Hu F (2022). Metabolic efficacy of time-restricted eating in adults: a systematic review and meta-analysis of randomized controlled trials. J Clin Endocrinol Metab.

[41] Moon S, Kang J, Kim SH, Chung HS, Kim YJ, Yu JM, Cho ST, Oh CM, Kim T (2020). Beneficial effects of time-restricted eating on metabolic diseases: a systemic review and meta-analysis. Nutrients.

[42] Gill S, Panda S (2015). A Smartphone App Reveals Erratic Diurnal Eating Patterns in Humans that Can Be Modulated for Health Benefits. Cell Metab.

[43] Gupta NJ, Kumar V, Panda S (2017). A camera-phone based study reveals erratic eating pattern and disrupted daily eating-fasting cycle among adults in India. PLoS One.

[44] Chakradeo P, Rasmussen HE, Swanson GR, Swanson B, Fogg LF, Bishehsari F, Burgess HJ, Keshavarzian A (2022). Psychometric testing of a food timing questionnaire and food timing screener. Curr Dev Nutr.

[45] Gioia SC, Guirette M, Chen A, Tucker C, Gray BE, Vetter C, Garaulet M, Scheer F, Saxena R, Dashti HS (2022). How accurately can we recall the timing of food intake? a comparison of food times from recall-based survey questions and daily food records. Curr Dev Nutr.

