# Supplementary material for: Meal-timing patterns and chronic disease prevalence in two representative Austrian studies
Source: Eur J Nutr. 2023 Mar 2;62(4):1879–90. doi: 10.1007/s00394-023-03113-z (PMC9980854; doi:10.1007/s00394-023-03113-z)
Supplement: Supplementary file 1 — Supplementary file1 (DOCX 234 KB) [file 394_2023_3113_MOESM1_ESM.docx]

**Supplementary table 1.** Sociodemographic characteristics across surveys in the total sample and among participants allocated to cluster groups.

|  | **Participants included in description of meal-timing** | | **Participants included in cluster solution** | |
| --- | --- | --- | --- | --- |
|  | **2017**  **(n=951)** | **2020**  **(n=952)** | **2017**  **(n=924)** | **2020**  **(n=870)** |
|  | ***N*(%)** | ***N*(%)** | ***N*(%)** | ***N*(%)** |
| **Age** |  |  |  |  |
| **18-24** | 123 (12.9) | 124 (13.0) | 119 (12.9) | 108 (12.4) |
| **25-34** | 164 (17.2) | 178 (18.7) | 161 (17.4) | 162 (18.6) |
| **35-44** | 220 (23.1) | 219 (23.0) | 210 (22.7) | 196 (22.5) |
| **45-54** | 244 (25.7) | 233 (24.5) | 238 (25.8) | 216 (24.8) |
| **≥55** | 200 (21.0) | 198 (20.8) | 196 (21.2) | 188 (21.6) |
| **Sex (Women)** | 487 (51.2) | 491 (51.6) | 473 (51.2) | 456 (52.4) |
| **BMI median(IQR)** | 24.7 (21.9-27.9) | 24.5 (21.6-28.0) | 24.7 (21.9-28.0) | 24.5 (21.6-28.0) |
| **Education** |  |  |  |  |
| **High school or less** | 385 (40.5) | 335 (35.2) | 371 (40.2) | 301 (34.6) |
| **Matura** | 351 (36.9) | 347 (36.5) | 344 (37.2) | 322 (37.0) |
| **University degree or above** | 215 (22.6) | 270 (28.4) | 209 (22.6) | 247 (28.4) |
| **Marital status** |  |  |  |  |
| **Single** | 288 (30.3) | 325 (34.1) | 279 (30.2) | 288 (33.1) |
| **Married/ in a partnership** | 540 (56.8) | 531 (55.8) | 525 (56.8) | 491 (56.4) |
| **Divorced** | 107 (11.3) | 81 (8.5) | 104 (11.3) | 77 (8.9) |
| **Widowed** | 16 (1.7) | 15 (1.6) | 16 (1.7) | 14 (1.6) |
| **Work status** |  |  |  |  |
| **Employed full time** | 484 (50.9) | 486 (51.1) | 468 (50.6) | 449 (51.6) |
| **Employed part time** | 106 (11.1) | 120 (12.6) | 103 (11.1) | 111 (12.8) |
| **Retired** | 126 (13.2) | 103 (10.8) | 121 (13.1) | 95 (10.9) |
| **Unemployed and disabled** | 70 (7.4) | 90 (9.5) | 69 (7.5) | 75 (8.6) |
| **Student, further training…** | 106 (11.1) | 121 (12.7) | 104 (11.3) | 108 (12.4) |
| **Household** | 59 (6.2) | 32 (3.4) | 59 (6.4) | 32 (3.7) |
| **Area of residence** |  |  |  |  |
| **Urban** | 435 (45.7) | 477 (50.1) | 426 (46.1) | 440 (50.6) |
| **Rural <50.000 inhabitants** | 402 (42.3) | 343 (36.0) | 389 (42.1) | 310 (35.6) |
| **Rural >50.000 *inhabitants*** | 114 (12.0) | 132 (13.9) | 109 (11.8) | 120 (13.8) |
| **Drinking alcohol** |  |  |  |  |
| **No standard glasses** | 381 (40.1) | 372 (39.1) | 368 (39.8) | 339 (39.0) |
| **1-6 standard glasses/week** | 309 (32.5) | 419 (44.0) | 301 (32.6) | 385 (44.3) |
| **7-12 standard glasses/week** | 134 (14.1) | 92 (9.7) | 131 (14.2) | 85 (9.8) |
| **>12 standard glasses/week** | 127 (13.3) | 69 (7.3) | 124 (13.4) | 61 (7.0) |
| **Smoking status** |  |  |  |  |
| **No, never** | 410 (43.1) | 424 (44.5) | 398 (43.1) | 384 (44.1) |
| **No, not anymore** | 258 (27.1) | 250 (26.3) | 247 (26.7) | 232 (26.7) |
| **Yes, I currently smoke** | 283 (29.8) | 278 (29.2) | 279 (30.2) | 254 (29.2) |
| **Time of physical activity^a^** |  |  |  |  |
| **No physical activity** | 354 (37.2) | 249 (26.2) | 343 (37.1) | 222 (25.5) |
| **Before 12pm** | 111 (11.7) | 153 (16.1) | 104 (11.3) | 147 (16.9) |
| **12.00-18.00** | 219 (23.0) | 237 (24.9) | 213 (23.1) | 214 (24.6) |
| **After 18.00** | 267 (28.1) | 313 (32.9) | 264 (28.6) | 287 (33.0) |
| **Self-rated chronotype^*^** |  |  |  |  |
| **Definetely a morning person** | 186 (19.6) | 202 (21.2) | 184 (19.9) | 189 (21.7) |
| **Rather a morning person** | 267 (28.1) | 256 (26.9) | 258 (27.9) | 235 (27.0) |
| **Rather an evening person** | 313 (32.9) | 273 (28.7) | 303 (32.8) | 250 (28.7) |
| **Definetely an evening person** | 185 (19.5) | 221 (23.2) | 179 (19.4) | 196 (22.5) |
| **Ever worked on nightshifts** | 250 (26.3) | 315 (33.1) | 250 (27.1) | 284 (32.6) |
|  |  |  |  |  |

a. In the survey in 2017 information on moderate and vigorous physical activity is available; in 2020 only on vigorous physical activity

**Supplementary figure 1.** Reported timing of main meals and snack after dinner on weekdays and weekends in **A)** 2017 and **B)** 2020.

**Supplementary figure 2. A)** Nighttime fasting interval, **B)** last meal to bed time and **C)** number of eating occasions in subjects eating and skipping breakfast during the week in the 2017 survey.

*Significant Pearson´s correlation coefficient (p<0.05).

**Supplementary figure 3. A)** Nighttime fasting interval, **B)** last meal to bed time and **C)** number of eating occasions in subjects eating and skipping breakfast during the week in the 2020 survey.

*significant Pearson´s correlation coefficient (p<0.05).

|  | | Survey 2017 (n=924) | | | Survey 2017 (n=870) | | |
| --- | --- | --- | --- | --- | --- | --- | --- |
|  |  | **Cluster A17**  **(n=720)**  **N(%)** | **Cluster B17 (n=204)**  **N(%)** | **AOR (95% CI)^a^** | **Cluster A20**  **(n=576)**  **N(%)** | **Cluster B20**  **(n=294)**  **N(%)** | **AOR (95% CI)^a^** |
| Chronic insomnia | |  |  |  |  |  |  |
|  | **Women** | 25 (6.6) | 14 (14.4) | 2.41 (1.20-4.86) | 21 (7.0) | 13 (8.2) | 1.17 (0.57-2.41) |
|  | **Men** | 18 (5.2) | 13 (12.1) | 2.63 (1.24-5.62) | 11 (4.0) | 10 (10.3) | 2.78 (1.22-6.32) |
| Depression | |  |  |  |  |  |  |
|  | **Women** | 39 (10.4) | 17 (17.5) | 1.87 (1.01-3.49) | 38 (12.8) | 34 (21.5) | 1.91 (1.15-3.19) |
|  | **Men** | 41 (11.9) | 12 (11.2) | 0.92 (0.46-1.83) | 27 (9.7) | 22 (16.2) | 1.77 (0.97-3.25) |
| Obesity | |  |  |  |  |  |  |
|  | **Women** | 55 (14.6) | 13 (13.4) | 0.87 (0.45-1.68) | 34 (11.4) | 28 (17.7) | 1.62 (0.93-2.80) |
|  | **Men** | 62 (18.0) | 22 (20.6) | 1.39 (0.79-2.44) | 57 (20.5) | 32 (23.5) | 1.14 (0.69-1.90) |
| Diabetes | |  |  |  |  |  |  |
|  | **Women** | 6 (1.6) | 1 (1.0) | 0.62 (0.07-5.26) | 8 (2.7) | 4 (2.5) | 0.90 (0.27-3.06) |
|  | **Men** | 27 (7.8) | 9 (8.4) | 1.35 (0.60-3.04) | 17 (6.1) | 10 (7.4) | 1.16 (0.51-2.67) |
| Hypertension | |  |  |  |  |  |  |
|  | **Women** | 38 (10.1) | 8 (8.3) | 0.75 (0.33-1.72) | 25 (8.4) | 22 (13.9) | 1.71 (0.90-3.22) |
|  | **Men** | 68 (19.8) | 17 (15.9) | 0.92 (0.50-1.70) | 52 (18.7) | 27 (19.9) | 1.02 (0.60-1.75) |
| Bad or very bad self-rated health status | |  |  |  |  |  |  |
|  | **Women** | 16 (4.3) | 12 (12.4) | 3.16 (1.44-6.94) | 9 (3.0) | 14 (8.9) | 3.02 (1.27-7.18) |
|  | **Men** | 22 (6.4) | 10 (9.3) | 1.69 (0.76-3.74) | 11 (4.0) | 14 (10.3) | 2.73 (1.20-6.20) |

**Supplementary table 2.** Association of meal timing behavior and self-rated health status and chronic disease in 2017 and 2020 stratified by sex categories. (*OR: Odds Ratio*, 95%CI: 95% confidence interval)

Multivariable adjusted *ORs* could not be calculated because of low numbers resulting after stratification. a. *AOR:* Adjusted *OR*. Adjusted for age. Calculated using unconditional logistic regression.

**Supplementary table 3.** Association of meal timing behavior and self-rated health status and chronic disease in 2017 and 2020 stratified by self-rated chronotype categories.

Multivariable adjusted *ORs* could not be calculated because of low numbers resulting after stratification. a. *AOR*: Adjusted *OR*. Adjusted for age and sex. Calculated using unconditional logistic regression. c. Morning or rather morning chronotype. d. Evening or rather evening chronotype.

|  |  |  |  |  |  |  |  |
| --- | --- | --- | --- | --- | --- | --- | --- |
|  | | **Survey 2017 (n=924)** | | | **Survey 2017 (n=870)** | | |
|  |  | **Cluster A17**  **(n=720)**  **N(%)** | **Cluster B17 (n=204)**  **N(%)** | **AOR (95% CI)^a^** | **Cluster A20**  **(n=576)**  **N(%)** | **Cluster B20**  **(n=294)**  **N(%)** | **AOR (95% CI)^a^** |
| Chronic insomnia | |  |  |  |  |  |  |
|  | **Early chronotype^c^** | 21 (5.7) | 10 (13.3) | 2.55 (1.15-5.68) | 16 (5.4) | 9 (7.1) | 1.29 (0.55-3.00) |
|  | **Late chronotype^d^** | 22 (6.2) | 12 (13.2) | 2.31 (1.18-4.52) | 16 (5.7) | 18 (10.8) | 1.98 (0.98-4.00) |
| Depression | |  |  |  |  |  |  |
|  | **Early chronotype^c^** | 29 (7.9) | 11 (14.7) | 2.01 (0.95-4.22) | 33 (11.1) | 26 (20.5) | 1.98 (1.12-3.50) |
|  | **Late chronotype^d^** | 51 (14.4) | 18 (14.0) | 0.96 (0.54-1.73) | 32 (11.5) | 30 (18.0) | 1.70 (0.99-2.92) |
| Obesity | |  |  |  |  |  |  |
|  | **Early chronotype^c^** | 66 (18.0) | 15 (20.0) | 1.14 (0.61-2.15) | 47 (15.8) | 21 (16.5) | 1.05 (0.59-1.89) |
|  | **Late chronotype^d^** | 51 (14.4) | 20 (15.5) | 1.16 (0.65-2.07) | 44 (15.8) | 39 (23.4) | 1.57 (0.96-2.56) |
| Diabetes | |  |  |  |  |  |  |
|  | **Early chronotype^c^** | 23 (6.3) | 5 (6.7) | 1.31 (0.46-3.76) | 10 (3.4) | 6 (4.7) | 1.49 (0.52-4.27) |
|  | **Late chronotype^d^** | 10 (2.8) | 5 (3.9) | 1.38 (0.45-4.24) | 15 (5.4) | 8 (4.8) | 0.79 (0.32-1.95) |
| Hypertension | |  |  |  |  |  |  |
|  | **Early chronotype^c^** | 64 (17.4) | 7 (9.3) | 0.49 (0.21-1.17) | 37 (12.5) | 18 (14.2) | 1.17 (0.61-2.22) |
|  | **Late chronotype^d^** | 42 (11.9) | 18 (14.0) | 1.27 (0.69-2.36) | 40 (14.3) | 31 (18.6) | 1.28 (0.75-2.19) |
| Bad or very bad self-rated health status | |  |  |  |  |  |  |
|  | **Early chronotype^c^** | 20 (5.4) | 7 (9.3) | 1.79 (0.73-4.41) | 10 (3.4) | 9 (7.1) | 2.10 (0.82-5.33) |
|  | **Late chronotype^d^** | 18 (5.1) | 15 (11.6) | 2.51 (1.22-5.17) | 10 (3.6) | 19 (11.4) | 3.35 (1.51-7.43) |

**Supplementary table 4.** Association of meal timing behaviour and self-rated health status and chronic disease in 2017 and 2020 in those who never worked on nightshifts. (*OR: Odds Ratio*, 95%CI: 95% confidence interval)

|  | **Survey 2017 (n=607)^a^** | | | **Survey 2020 (n=552)^b^** | | |
| --- | --- | --- | --- | --- | --- | --- |
|  | **Cluster A17**  **(n=474)**  **N(%)** | **Cluster B17 (n=133)**  **N(%)** | **AOR (95% CI)^c^** | **Cluster A20**  **(n=371)**  **N(%)** | **Cluster B20**  **(n=181)**  **N(%)** | **AOR (95% CI)^c^** |
| **Chronic insomnia** | 23 (4.9) | 13 (9.8) | 2.15 (1.05-4.38) | 18 (4.9) | 12 (6.6) | 1.40 (0.66-2.98) |
| **Depression** | 39 (8.2) | 18 (13.5) | 1.79 (0.99-3.27) | 36 (9.7) | 23 (12.7) | 1.37 (0.78-2.39) |
| **Obesity** | 59 (12.4) | 23 (17.3) | 1.49 (0.87-2.56) | 52 (14.0) | 28 (15.5) | 1.09 (0.66-1.81) |
| **Diabetes** | 12 (2.5) | 8 (6.0) | 2.50 (0.97-6.45) | 10 (2.7) | 6 (3.3) | 1.18 (0.42-3.33) |
| **Hypertension** | 56 (11.8) | 16 (12.0) | 1.04 (0.56-1.93) | 40 (10.8) | 23 (12.7) | 1.17 (0.67-2.03) |
| **Bad or very bad self-rated health status** | 15 (3.2) | 11 (8.3) | 2.74 (1.22-6.14) | 11 (3.0) | 13 (7.2) | 2.51 (1.10-5.73) |

Multivariable adjusted *ORs* could not be calculated because of low numbers resulting after excluding previous nightshift workers.

a.n=317 participants who reported having worked on nightshift or with unknown nightshift history were exluded. b. n=318 who reported having worked on nightshift or with unknown nightshift history were exluded. c. *AOR:* Adjusted *OR*. Adjusted for age and sex. Calculated using unconditional logistic regression.

**Supplementary table 5.** Association of meal timing behaviour and self-rated health status and chronic disease in 2017 and 2020 in those drinking 6 alcohol standard glasses per week or less. (OR: Odds Ratio, 95%CI: 95% confidence interval)

|  | **Survey 2017 (n=669)^a^** | | | **Survey 2020 (n=724)^b^** | | |
| --- | --- | --- | --- | --- | --- | --- |
|  | **Cluster A17**  **(n=530)**  **N(%)** | **Cluster B17 (n=139)**  **N(%)** | **AOR (95% CI)^c^** | **Cluster A20**  **(n=485)**  **N(%)** | **Cluster B20**  **(n=239)**  **N(%)** | **AOR (95% CI)^c^** |
| **Chronic insomnia** | 33 (6.2) | 20 (14.4) | 2.58 (1.43-4.68) | 28 (5.8) | 19 (7.9) | 1.40 (0.76-2.56) |
| **Depression** | 60 (11.3) | 18 (13.0) | 1.17 (0.66-2.06) | 52 (10.7) | 46 (19.2) | 1.98 (1.29-3.06) |
| **Obesity** | 86 (16.2) | 29 (20.9) | 1.38 (0.85-2.22) | 74 (15.3) | 48 (20.1) | 1.40 (0.93-2.12) |
| **Diabetes** | 21 (4.0) | 8 (5.8) | 1.64 (0.67-3.99) | 21 (4.3) | 13 (5.4) | 1.30 (0.63-2.68) |
| **Hypertension** | 56 (10.6) | 17 (12.2) | 1.20 (0.66-2.19) | 62 (12.8) | 35 (14.6) | 1.18 (0.74-1.90) |
| **Bad or very bad self-rated health status** | 30 (5.7) | 16 (11.5) | 2.17 (1.14-4.13) | 18 (3.7) | 21 (8.8) | 2.49 (1.30-4.79) |

Multivariable adjusted *ORs* could not be calculated because of low numbers resulting after excluding participants drinking more than 6 standard glasses a week. a. n=255 participants who reported drinking more than 6 alcohol standard glasses per week were exluded. b.n=146 participants who reported drinking more than 6 alcohol standard glasses per week were exluded. c. *AOR*: Adjusted *OR*. Adjusted for age and sex. Calculated using unconditional logistic regression.
